# *In Vivo* Antimalarial Activity of 80% Methanol and Aqueous Bark Extracts of *Terminalia brownii* Fresen. (Combretaceae) against *Plasmodium berghei* in Mice

**DOI:** 10.1155/2020/9749410

**Published:** 2020-01-22

**Authors:** Hana Biruk, Biruk Sentayehu, Yonatan Alebachew, Wondmagegn Tamiru, Abebe Ejigu, Solomon Assefa

**Affiliations:** ^1^Department of Pharmacology and Clinical Pharmacy, School of Pharmacy, Addis Ababa University, Addis Ababa, Ethiopia; ^2^Department of Pharmaceutical Chemistry and Pharmacognosy, School of Pharmacy, Addis Ababa University, Addis Ababa, Ethiopia

## Abstract

**Background:**

Despite a substantial scientific progress over the past two decades, malaria continues to be a worldwide burden. Evergrowing resistance towards the currently available antimalarial drugs is a challenge to combat malaria. Medicinal plants are a promising source of new drugs to tackle this problem. Thus, the present study aimed at evaluating the antiplasmodial activity of *Terminalia brownii* in *Plasmodium berghei* infected mice.

**Methods:**

A 4-day suppressive test was employed to evaluate the antimalarial effect of 80% methanol and aqueous bark extracts of *T. brownii* against *P. berghei* in Swiss albino mice.

**Results:**

The *in vivo* acute toxicity test indicated that both extracts of *T. brownii* against *p* < 0.001) compared to negative control. The maximum level of chemosuppression (60.2%) was exhibited at 400 mg/kg dose of 80% methanol extract. Moreover, the 80% methanol extract showed a significant (*p* < 0.001) compared to negative control. The maximum level of chemosuppression (60.2%) was exhibited at 400 mg/kg dose of 80% methanol extract. Moreover, the 80% methanol extract showed a significant (

**Conclusion:**

The present study indicated that hydromethanolic and aqueous bark extracts of *T. brownii* possess a promising antimalarial activity, with higher effect exhibited by the hydromethanolic extract.*T. brownii* against

## 1. Background

Malaria is a life-threatening disease caused by *Plasmodium* parasites that are transmitted to human through the bites of infected female *Anopheles* mosquitoes [1]. Four species of *Plasmodium* have long been recognized to infect humans in nature. In addition, there is one species, *P. knowlesi*, that naturally infects macaques and has recently been recognized to be a cause of zoonotic malaria in humans [2, 3].

According to the World Health Organization's Malaria Report, there were 216 million cases of malaria in 2016. The estimated number of malaria deaths stood at 445,000 in the same year. In 2016, the African region was home to 90% of malaria cases and 91% of malaria deaths [1]. Moreover, malaria remains a major killer of children under five years, taking the life of a child every two minutes [1]. It is also a severe disease in Ethiopia, where 75% of the land is malarious and more than 54 million people are vulnerable [4, 5].

Despite a substantial scientific progress over the past two decades, malaria continues to be a worldwide burden [6]. Tremendous effort has been made in the fight against malaria using core disease prevention tools, prompt diagnosis, and treatment with antimalarial medicines. However, the rate at which incidence and mortality of malaria declines has stalled and even reversed in some regions [7]. The resistance towards the currently available antimalarial drugs is seriously challenging the effort to combat malaria.

Constituting a promising source of new drugs, medicinal plants have been given a priority interest worldwide in the search of safe and effective antiplasmodial agents from plants [8]. Artemisinin derivatives and cinchona alkaloids, such as quinine, are quite exemplary of such assertions. Ethiopia is endowed with abundant medicinal plant resources and traditional medicinal practices [9]. Studies conducted on several traditionally claimed Ethiopian medicinal plants confirmed their antimalarial activities [10–16].

Accordingly, the present study aimed at investigating the *in vivo* antimalarial activity of the most important plant used in the Ethiopian folkloric medicine for the treatment of malaria, *Terminalia brownii* [17]. It is a leafy deciduous tree that is usually 4–15 m high with a rounded, flat topped, spreading crown, and a straight trunk. In a recent study [18], two compounds with a galloyl group, isolated from stem bark extract of the plant, exhibited *in vitro* antiplasmodial activity against chloroquine-sensitive and chloroquine-resistant strains of *P. falciparum.* But, *in vivo* antimalarial activity of *T. brownii* bark was not investigated. The present study, thus, evaluated the *in vivo* antimalarial activity of 80% methanol and aqueous extracts of *T. brownii* barks against *P. berghei* in mice.

## 2. Materials and Methods

### 2.1. Collection and Authentication of Plant Material

Fresh barks of *T. brownii* Fresen. were collected from Harar town, Eastern Ethiopia, in August 2017. The collected plant material was authenticated by a taxonomist at the National Herbarium, Addis Ababa University, department of biology, where a voucher specimen (HB001) is kept for future reference.

### 2.2. Experimental Animals

Healthy Swiss albino mice (22–31 g), aged 6–8 weeks (female for toxicity and male for the study), were purchased from the Ethiopian Public Health Institute (EPHI) and kept in the animal house at the School of Pharmacy, Addis Ababa University. Animals were acclimatized for a week just before the experimentation began. Animals were housed in polyethylene cages at room temperature with a 12 h light-dark cycle and provided with a commercial food and water ad libitum. All procedures and techniques used in this study were in accordance with the Guide for Care and Use of Laboratory Animals [19].

### 2.3. Rodent Parasite

Chloroquine-sensitive strain of *Plasmodium berghei* (ANKA) was obtained from EPHI for the *in vivo* antimalarial assay. The parasites were maintained by serial passage of blood from infected mice to noninfected ones on a weekly basis.

### 2.4. Extraction

The collected barks of the plant were thoroughly washed with tap water and cleaned with gauze to remove dirt and soil. The manually ground pieces of the bark were dried under shade and pulverized using a mortar and pestle to get a coarse powder before the extraction. The powdered plant material (600 g) was evenly divided into two portions, for 80% methanol and aqueous extraction.

The first portion (300 g) was extracted by cold maceration (100 g of powder in 500 ml of 80% methanol). The maceration was facilitated using an orbital shaker at 145 rpm for 72 h. The mixture was filtered using a cotton cloth. The filtrates were further passed through Whatman filter paper (No. 1). The residues were remacerated twice, using the same procedure. The methanol in the filtrate of the hydromethanolic extract was removed under reduced pressure by a rotary evaporator (Buchi type TRE121, Switzerland) at 45 rpm and 40°C to obtain 80% methanol extract. The extract was further concentrated to dryness with a lyophilizer (Wagtech Jouan Nordic DK-3450 Allerod, Denmark) at −50°C and under reduced pressure (200 mBar). At the end of the procedure, a 60 g of dried crude hydromethanolic extract (20% yield) was harvested.

For aqueous extraction, bark powder (300 g) were boiled in 1500 ml of distilled water for an hour. The mixture was then filtered using muslin cloth, and filtrates were passed through Whatman filter paper (No. 1). To exhaustively extract the plant part, the procedure was repeated twice. The aqueous extract was frozen in a deep freezer overnight and then freeze-dried with a lyophilizer, using similar conditions stated above. The aqueous extraction yielded 54 g of dried extract (18% yield). Both extracts were stored in screw cap vials in a refrigerator at −4°C until use.

### 2.5. Acute Toxicity Study and Dose Selection

The 80% methanol and aqueous extracts of *T. Brownii* were evaluated for their acute toxicity in noninfected female Swiss albino mice, according to OECD Guideline [20]. For each extract, five female mice were used. All mice were fasted for 4 h before and 2 h after the administration of the extract. First, a sighting study was performed to determine the starting dose in one mouse. For this, a single dose of 2,000 mg/kg was administered orally. No death was observed within 24 h. Therefore, an additional four mice were given the same dose of the extract. The animals were observed for overt toxicities such as diarrhea, weight loss, tremor, lethargy, and paralysis periodically for the first four hours during the 24 h period and then followed for 14 days for any lethality. After acute toxicity test, three dose levels (100 mg/kg, 200 mg/kg, and 400 mg/kg) were selected for both extracts [20].

### 2.6. Parasite Inoculation

To infect the mice, blood from a donor mouse with a parasitemia of about 32–37% was collected in a Petri dish containing trisodium citrate as an anticoagulant [21]. The blood was then diluted in normal saline so that the final suspension would contain about 1 × 10^7^ infected RBCs in every 0.2 ml suspension. Therefore, each mouse used was infected intraperitoneally with 0.2 ml of infected blood containing about 1 × 10^7^*P. berghei* parasitized RBCs.

### 2.7. Grouping and Dosing of Animals

*P. berghei* infected mice were randomly divided into eight groups of five mice per group. Group I received 0.2 ml/kg of distilled water and served as a negative control. Group II received 25 mg/kg dose of chloroquine (CQ), the standard antimalarial drug. Groups III, IV, and V were treated with 80% methanol extract at a dose of 100 mg/kg, 200 mg/kg, and 400 mg/kg, respectively. The remaining groups, groups VI, VII, and VIII, were treated with aqueous extract at 100 mg/kg, 200 mg/kg, and 400 mg/kg doses, respectively. Each dose was reconstituted by distilled water and administered orally using oral gavage.

### 2.8. Four-Day Suppressive Test

Evaluation of schizonticidal activity of the plant on early infection was carried out according to the method described by Peter et al. [22]. Treatment, as described in Section 2.7, was started two hours after the mice were inoculated with the parasite on Day 0. Treatment was then continued daily for four days (from Day 0 to Day 3). On 5^th^ day (D4), blood was collected from the tail of each mouse using clean, nongreasy slides, and thin blood films were made accordingly. The air-dried blood films were then fixed with few drops of methanol and stained with Giemsa at a pH of 7.2. Parasitemia was examined using a light microscope (Olympus N-120A, Philippines) with X100 objective for determining blood parasite suppression. In addition, each mouse was attended daily for the determination of survival time after treatment. Packed cell volume (PCV) was recorded just before infection and at the end of the experiment.

#### 2.8.1. Parasitemia and Survival Time Measurement

The percentage parasitemia was obtained by counting the number of parasitized red blood cells (PRBC) out of the total erythrocytes in random fields of the microscope. Two stained slides for each mouse were examined using the formula as follows [23]:

(1) $$\begin{array}{c} \%\text{ parasitemia}=\frac{\text{number of PRBC}}{\text{total number of RBC}}*\;100\%. \end{array}$$

Percent parasitemia suppression was calculated using the following formula [23]:

(2) $$\begin{array}{c} \text{average }\%\text{ suppression of parasitemia }=\frac{\text{parasitemia in negative control}-\text{parasitemia in treatment group}}{\text{parasitemia in negative control}}\times \;100. \end{array}$$

Mice were monitored daily, and the number of days from the time of inoculation up to death was recorded for each mouse in treatment and control groups throughout the follow-up period. The mean survival time (MST) for each group was calculated as follows [14]:

(3) $$\begin{array}{c} \text{MST}=\frac{\text{sum of survival time of all mice in a group }\left(\text{days}\right)}{\text{total number of mice in that group}}. \end{array}$$

#### 2.8.2. Packed Cell Volume Measurement

PCV was measured to assess the effect of the two extracts in preventing the hemolytic effect of the *Plasmodium* parasite. Heparinized capillary tubes were used for the collection of blood from tail of the mice. The tubes were filled to 3/4^th^ of their height with blood and sealed with sealing clay at their dry end. The tubes were then placed on a microhematocrit centrifuge (Centurion Scientific, UK) with the sealed end facing the periphery and centrifuged at 11,000 rpm for 5 min. Finally, PCV was determined using a standard hematocrit reader (Hawksley and Sons, England) [22].

(4) $$\begin{array}{c} \text{PCV}=\frac{\text{volume of erythrocytes in a given volume of blood}}{\text{total blood volume examined}}\times 100\%\text{.} \end{array}$$

#### 2.8.3. Body Weight and Rectal Temperature Measurement

Each mouse in a group was weighed using sensitive digital weighing balance, and rectal temperature was measured using digital rectal thermometer. The percentage changes of their mean values that occurred before and after treatment were then calculated.

### 2.9. Preliminary Phytochemical Analysis

Both extracts were screened for the presence of different phytochemical constituents following standard procedures [24].

### 2.10. Data Analysis

Results of the study are expressed as mean ± standard error of mean (SEM). Data were analyzed using SPSS, version 20. Statistical significance was determined by one-way analysis of variance (ANOVA) followed by Tukey post hoc test to compare the levels of parasitemia, survival time, and changes in body weight, PCV, and rectal temperature of the *P. berghei* infected mice between the control and extract-treated groups. *p* values <0.05 were considered as statistically significant.

## 3. Results

### 3.1. Acute Toxicity Study

In the *in vivo* acute toxicity test at a limit dose of 2,000 mg/kg, both extracts of *T. brownii* did not cause mortality and body weight reduction within the first 24 h as well as in the subsequent 14 days. Moreover, gross physical and behavioral observational study revealed no visible signs of toxicity such as lacrimation, hair erection, and reduction in their motor and feeding activities during the 14 days' follow-up.

### 3.2. Chemosuppressive Effect of the Plant

The 4-day chemosuppressive study revealed that the 80% methanol and aqueous extracts of *T. brownii* exhibited a significant inhibition of parasitemia (*p* < 0.001) in a dose-dependent manner compared to negative control (Table 1). The level of suppression revealed by 80% methanol extract at concentrations of 100 mg/kg/day, 200 mg/kg/day, and 400 mg/kg/day in the 4-day suppressive test was 32.7%, 47.1%, and 60.2%, respectively. Moreover, the 400 mg/kg dose of 80% methanol extract showed a significant inhibition of parasitemia (*p* < 0.001) compared to the 200 mg/kg and 100 mg/kg doses of the same extract. The aqueous extract, on the other hand, exhibited a percent inhibition of 25.9%, 39.4%, and 51.1% at dose levels of 100 mg/kg/day, 200 mg/kg/day, and 400 mg/kg/day, respectively.

The 80% methanol and aqueous extracts exhibited a dose-dependent significant (*p* < 0.001) increment of MST compared to negative control. However, their capability of increasing survival time was smaller than that of the standard chloroquine.

The 80% methanol extract at concentration of 200 mg/kg/day and 400 mg/kg/day significantly (*p* < 0.001) prevented loss of body weight associated with infection in a dose-dependent manner compared to control (Table 2). The standard (chloroquine) provided a significant (*p* < 0.001) protection compared to all doses of the 80% methanol as well as the aqueous extracts.

As indicated in Table 3, the 80% methanol extract was able to significantly (*p* < 0.001) avert lowering of rectal temperature associated with infection in a dose-dependent manner. The aqueous extract, however, exhibited reduction in rectal temperature associated with infection.

**Figure 1:**
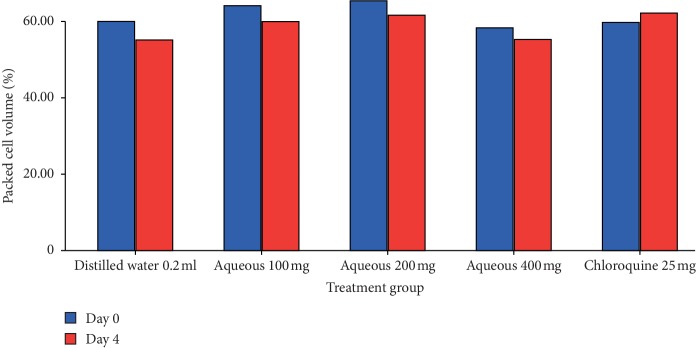
The effect of aqueous extract of *T. brownii* on packed cell volume of *P. berghei* infected mice on 4-day suppressive test. Values are presented as mean ± SEM; *n* = 5; aqueous refers to aqueous extract; numbers refer to doses in mg/kg/day.

**Figure 2:**
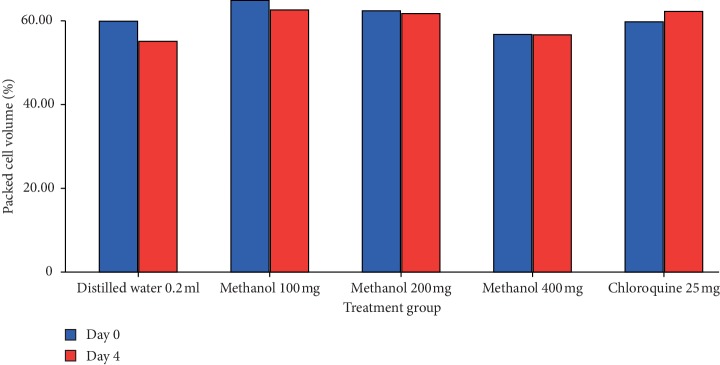
The effect of 80% methanol extract of *T. brownii* on packed cell volume of *P. berghei* infected mice on 4-day suppression test. Values are presented as mean ± SEM; *n* = 5; methanol refers to methanol extract; numbers refer to doses in mg/kg/day.

The 80% methanol extract showed a significant (*p* < 0.001) attenuation of anemia associated with infection in a dose-dependent manner compared to control (Figure 2). Even though the aqueous extract showed a negative percentage change of PCV (Figure 1), it significantly (*p* < 0.001) showed attenuation of anemia associated with infection compared to negative control. Chloroquine exhibited a significant (*p* < 0.001) attenuation of anemia associated with infection compared to all doses of the 80% methanol as well as the aqueous extract.

### 3.3. Preliminary Phytochemical Analysis

Phytochemical screening of 80% methanol and aqueous extracts of *T. brownii* bark revealed the presence of flavonoids, saponins, steroids, tannins, and terpenoids in both extracts (Table 4). However, anthraquinone (o-glycoside) is only present in 80% methanol extract.

## 4. Discussion

Malaria remains a major public health problem in the world, which is responsible for death of millions of people, particularly in sub-Saharan Africa. Nowadays, malaria control has gradually become more complex due to the spread of drug-resistant parasites [1]. To foil such challenges, it is essential to do extensive researches directed towards the search for new antimalarial drugs. In this regard, medicinal plants have a remarkable track record as a source of safe and effective antimalarial drugs. Artemisinin derivatives could be cited as a prominent example to indicate medicinal plants role in the drug discovery of antimalarial agents. Among several medicinal plants, *T. Brownii* is one of medicinal plants of antimalarial use in Ethiopian folkloric medicine [25].

The *in vivo* evaluation of antimalarial compound(s) begins with the use of rodent malaria parasites [26]. Mouse models have demonstrated utility in delineating the mechanisms of several conventional antimalarial agents [21]. *P. berghei* (ANKA) has been used in studying the activity of antimalarials in mice. This is mainly because of its ability to produce a rodent model of malaria that is similar to human malaria infection [26]. Suppressive test using *P. berghei* infected mice provides a preclinical indication of potential bioactivity of the test sample [27, 28].

Oral dosing of the extracts was used in this study, to replicate the ethnomedical method of administration and the likely route during clinical evaluation [25, 29]. Moreover, the acute toxicity result of the present study suggested that the oral medial lethal dose (LD_50_) of both extracts is much greater than 2000 mg/kg as per OECD guideline No. 425 [20].

*In vivo* antiplasmodial activity can be classified as moderate, good, and very good if an extract displayed respective percent parasite suppression equal to or greater than 50% at doses of 500, 250, and 100 mg/kg body weight per day, respectively [30]. In this study, the level of suppression of 80% methanol extract (400 mg/kg/day) was 60.2%. Based on the above classification, the plant is considered to have exhibited a good antiplasmodial activity. This assertion is evidenced by other *in vivo* studies that reported antimalarial activity of other species of the same genus such as *T. chebula*, *T. bellerica* [31], and *T. macroptera* [32].

Recently, Mbouna et al. [33] reported the *in vitro* antiplasmodial activity of *T. mantaly* (IC_50_ = 0.26 *μ*g/mL) and *T. superba* (IC_50_ = 0.57 *μ*g/mL) against *P. falciparum*. Moreover, the parasite suppression exhibited by 80% methanol extracts is comparable to results of former studies conducted on methanol extract of *Artemisia abyssinica* [34], *Croton macrostac*hyus [11, 35], and *Strychnos mitis* [36]. The chemosuppression exhibited by aqueous extract, however, is lower than a study conducted on the aqueous extract of *S. mitis* [36].

As is revealed in phytochemical analysis, the presence of flavonoids, saponins, steroids, tannins and terpenoids in both extracts of the study plant could be responsible for its antimalarial activity [18]. The superior antimalarial effect of the methanolic extract could probably be related to the presence of anthraquinone (O-glycoside) in the 80% methanol extract but not in the aqueous extract [37].

Both extracts of the plant prolonged the mean survival time of the experimental mice indicating that the plant suppressed *P. berghei* and reduced the overall pathologic effect of the parasite on mice. Similar result on mean survival time of mice was reported in studies conducted on *T. chebula*, *T. bellerica* [31], and *T. macroptera* [32].

Anemia, body weight loss, and body temperature reduction are the general features of malaria-infected mice [37]. Thus, antimalarial agents are expected to prevent pathological features due to a rise in parasitemia. In this study, the 80% methanol extract exhibited a superior attenuation of anemia compared to the aqueous extract. This likely suggests that the presence of anthraquinone (O-glycoside) may be the major active component that contributes to the antimalarial activity of *T. brownii* [38].

## 5. Conclusion

The present study indicated a promising *in vivo* antiplasmodial effect of the 80% methanol and aqueous extracts of *T. brownii*. The extracts were also found to be nontoxic at the maximum tested dose of 2000 mg/kg. In addition to upholding the traditional claim of the plant, the results of this study provide a basis for investigating the active compounds of this plant with the purpose of discovering candidate compounds for the development of effective and safe antimalarial drugs.

## Figures and Tables

**Table 1:** Percent parasitemia and suppression of infected mice treated with 80% methanol extract and aqueous extracts of *Terminalia brownii* in the 4-day suppressive test.

Test substances	Dose (mg/kg/day)	% parasitemia	% suppression	Survival days
Distilled water	0.2 ml	25.5 ± 0.27	0.00	5.8 ± 0.20

Methanol extract	100 mg	17.2 ± 0.29	32.7^a^^*∗*^^c^^*∗*^^d^^*∗*^	8.0 ± 0.00^a^^*∗*^
	200 mg	13.5 ± 0.26	47.1^a^^*∗*^^b^^*∗*^^d^^*∗*^	10.8 ± 0.20^a^^*∗*^
	400 mg	10.2 ± 0.28	60.2^a^^*∗*^^b^^*∗*^^c^^*∗*^^g^^*∗*^	12.8 ± 0.20^a^^*∗*^^e^^*∗*^

Aqueous extract	100 mg	18.9 ± 0.49	25.9^a^^*∗*^^f^^*∗∗*^^g^^*∗*^	6.8 ± 0.20^a^^*∗*^
	200 mg	15.5 ± 0.22	39.4^a^^*∗*^^e^^*∗*^^g^^*∗*^	7.0 ± 0.00^a^^*∗*^
	400 mg	12.5 ± 0.27	51.1^a^^*∗*^^d^^*∗*^^e^^*∗*^^f^^*∗*^	7.4 ± 0.24^a^^*∗*^

Chloroquine	25 mg	0.0 ± 0.00	100.0^a^^*∗*^^b^^*∗*^^c^^*∗*^^d^^*∗*^^e^^*∗*^^f^^*∗*^^g^^*∗*^	28.0 ± 0.00^a^^*∗*^^b^^*∗*^^c^^*∗*^^d^^*∗*^^e^^*∗*^^f^^*∗*^^g^^*∗*^

Values are presented as *M* ± SEM; *n* = 5; ^*∗*^(*p* < 0.001); a = compared to negative control; b = compared to 80% methanol extract 100 mg; c = compared to 80% methanol extract 200 mg; d = compared to 80% methanol extract 400 mg; e = compared to aqueous extract 100 mg; f = compared to aqueous extract 200 mg; g = compared to aqueous extract 400 mg.

**Table 2:** Body weight of infected mice treated with 80% methanol and aqueous extracts of *Terminalia brownii* in the 4-day suppressive test.

Test substances	Dose (mg/kg/day)	Pretreatment body weight	Posttreatment body weight	Percentage change %
Distilled water	0.2 ml	27.1 ± 1.17	23.0 ± 1.08	−1.3

Methanol extract	100 mg	26.2 ± 0.67	24.2 ± 0.70	−0.4^a^^*∗*^
	200 mg	26.9 ± 0.53	26.9 ± 0.58	+0.2^a^^*∗*^^c^^*∗*^
	400 mg	26.9 ± 1.00	27.1 ± 1.09	+0.6^a^^*∗*^^c^^*∗*^

Aqueous extract	100 mg	29.1 ± 0.40	26.4 ± 0.43	−0.6^a^^*∗*^
	200 mg	30.5 ± 0.34	28.2 ± 0.33	−0.3^a^^*∗*^
	400 mg	28.9 ± 0.62	26.7 ± 0.64	−0.4^a^^*∗*^

Chloroquine	25 mg	26.9 ± 0.80	29.8 ± 0.80	+2.1^a^^*∗*^^bc^^*∗*^

Values are presented as *M* ± SEM; *n* = 5; ^*∗*^(*p* < 0.001); a = compared to negative control; b = compared to 80% methanol extract; c = compared to aqueous extract.

**Table 3:** Rectal temperature of infected mice treated with 80% methanol and aqueous extracts of *Terminalia brownii* in the 4-day suppressive test.

Test substances	Dose (mg/kg/day)	Pretreatment rectal temperature	Posttreatment rectal temperature	Percentage change %
Distilled water	0.2 ml	35.9 ± 0.34	33.3 ± 0.31	−0.4

Methanol extract	100 mg	35.8 ± 0.28	35.8 ± 0.22	+0.1^a^^*∗*^^c^^*∗∗*^^d^^*∗*^
	200 mg	36.5 ± 0.26	36.5 ± 0.23	+0.3^a^^*∗*^^c^^*∗∗*^^d^^*∗*^
	400 mg	37.0 ± 0.21	37.5 ± 0.32	+0.5^a^^*∗*^^c^^*∗∗*^^d^^*∗*^

Aqueous extract	100 mg	36.4 ± 0.24	33.3 ± 0.25	−0.5
	200 mg	36.9 ± 0.20	34.3 ± 0.19	−0.3
	400 mg	35.9 ± 0.17	33.4 ± 0.18	−0.4

Chloroquine	25 mg	36.2 ± 0.15	36.7 ± 0.12	+0.7^a^^*∗*^^b^^*∗*^^c^^*∗*^

Values are presented as *M* ± SEM; *n* = 5. ^*∗∗*^(*p* < 0.01); ^*∗*^(*p* < 0.001); a = compared to negative control; b = compared to 80% methanol extract; c = compared to aqueous extract; d = compared to chloroquine.

**Table 4:** Preliminary phytochemical screening of 80% methanol and aqueous extract of *Terminalia brownii* bark.

Phytoconstituents	80% methanol extract	Aqueous extract
Alkaloids	−	−
Anthraquinone (free)	−	−
Anthraquinone (O-glycoside)	+	−
Flavonoids	+	+
Cardiac glycosides	−	−
Coumarin	−	−
Saponins	+	+
Steroids	+	+
Tannins	+	+
Terpenoids	+	+

−, absent; +, present.

## Data Availability

The data used to support the findings of this study will be available from the corresponding author on a reasonable request.
